# Cell-independent matrix configuration in early corneal development

**DOI:** 10.1016/j.exer.2019.107772

**Published:** 2019-10

**Authors:** Robert D. Young, Carlo Knupp, Elena Koudouna, James R. Ralphs, Yanhui Ma, Peter Y. Lwigale, James V. Jester, Andrew J. Quantock

**Affiliations:** a Structural Biophysics Group, School of Optometry & Vision Sciences, Cardiff University, Maindy Road, Cathays, Cardiff, CF24 4HQ, Wales, UK; b School of Biosciences, Cardiff University, Sir Martin Evans Building, Museum Avenue, Cardiff, CF10 3AX, Wales, UK; c Department of Biomedical Engineering, Ohio State University, 1080 Carmack Road, Columbus, OH 43210, USA; d Department of Biosciences, Rice University, 6100 Main Street, Houston, TX, 77005, USA; e Departments of Ophthalmology and Biomedical Engineering, University of California, 843 Health Sciences Road, Irvine, CA, 92697, USA

**Keywords:** Cornea, Collagen, Development, Serial block face scanning electron microscopy, SBF SEM, serial block face scanning electron microscopy, SHG, second harmonic generation, E, embryonic day, CS/DS, chondroitin sulphate/dermatan sulphate

## Abstract

Mechanisms controlling the spatial configuration of the remarkably ordered collagen-rich extracellular matrix of the transparent cornea remain incompletely understood. We previously described the assembly of the emerging corneal matrix in the mid and late stages of embryogenesis and concluded that collagen fibril organisation was driven by cell-directed mechanisms. Here, the early stages of corneal morphogenesis were examined by serial block face scanning electron microscopy of embryonic chick corneas starting at embryonic day three (E3), followed by a Fourier transform analysis of three-dimensional datasets and theoretical considerations of factors that influence matrix formation. Eyes developing normally and eyes that had the lens surgically removed at E3 were studied. Uniformly thin collagen fibrils are deposited by surface ectoderm-derived corneal epithelium in the primary stroma of the developing chick cornea and form an acellular matrix with a striking micro-lamellar orthogonal arrangement. Fourier transform analysis supported this observation and indicated that adjacent micro-lamellae display a clockwise rotation of fibril orientation, depth-wise below the epithelium. We present a model which attempts to explain how, in the absence of cells in the primary stroma, collagen organisation might be influenced by cell-independent, intrinsic mechanisms, such as fibril axial charge derived from associated proteoglycans. On a supra-lamellar scale, fine cords of non-collagenous filamentous matrix were detected over large tissue volumes. These extend into the developing cornea from the epithelial basal lamina and appear to associate with the neural crest cells that migrate inwardly to form, first the corneal endothelium and then keratocytes which synthesise the mature, secondary corneal stroma. In a small number of experimental specimens, matrix cords were present even when periocular neural crest cell migration and corneal morphogenesis had been perturbed following removal of the lens at E3.

## Highlights

• Highly-ordered connective tissue appears early in development of the avian cornea.
• Cell-independent mechanisms may contribute to the organisation of collagen fibrils into an orthogonal array.
• Matrix cords from epithelium into stroma contact invading neural crest cells.

## Graphical abstract

**Figure:**
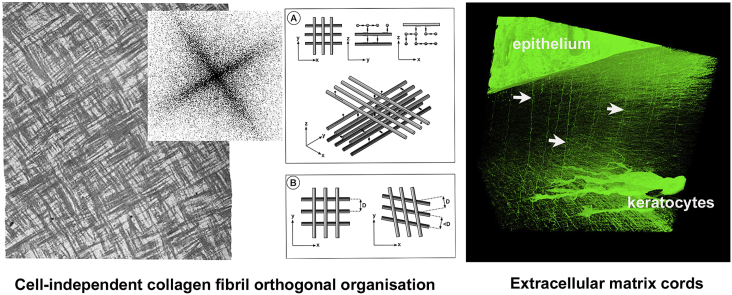

## 1 Introduction

The stroma of the cornea represents perhaps the most exquisite example in terms of its structural organisation of all connective tissues. For the majority of its thickness, collagen fibrils of regular, narrow diameter and close separation, form layers or lamellae, superimposed one upon another. Within each lamella fibrils exhibit parallel alignment, with a gradual clockwise displacement in orientation in the distal two thirds of the stroma, progressing from superficial to deeper locations (Trelstad and Coulombre, 1971; Koudouna et al., 2018a). These highly ordered features are generally accepted to have functional significance in relation to the transparency and biomechanical properties of the cornea (Quantock and Young, 2008; Hassell and Birk, 2010; Chen et al., 2015). An understanding of the mechanisms underpinning the biosynthesis of this remarkably organised assembly of macromolecules would also provide invaluable insights into the development of less homogeneous matrices in other tissues. Contemporarily, there is considerable interest in the mechanisms controlling biosynthesis of collagenous connective tissues with specialised functions, particularly those with lamellar structures (Ghazanfari et al., 2016), in order to gain useful guidelines for the generation of biomimetic substrates for clinical use in tissue reconstruction and repair. Consequently, events that direct the embryonic growth of the cornea continue to be intensively studied.

Investigations of corneal development have been carried out over more than fifty years the majority using as a model the embryonic chick cornea, which derives from an initial primary stroma produced by the ectoderm. This rudimentary matrix is generally believed to exert a controlling influence upon the alignment of presumptive keratocytes, which migrate into it, and also upon the organisation of the secondary mature stroma, which the keratocytes subsequently synthesise. However, it seems possible that the primary stroma might not fulfil an essential role in corneal morphogenesis when one considers that it is not present in embryonic mammalian cornea (Feneck et al., 2019). Further evidence of the complexity of mechanisms involved in early cell-matrix interactions comes from experimental studies showing that corneal cells *in vitro* are able to exhibit a tissue specific orthogonal alignment in the absence of directional cues (Doane and Birk, 1991). The primary stroma of the developing chick cornea consists of a fibrillar matrix of type I and type II collagens with minor amounts of associated type IX collagen (Svoboda et al., 1988; Fitch et al., 1995). Type II and type IX collagens are replaced by type I and type V as principal components of the secondary stroma. Multipotent periocular cells, originating in the neural crest, undergo distinctive alterations in gene expression as they migrate to form corneal endothelium and keratocytes (Bi and Lwigale, 2019). The latter synthesise the mature stroma, and there is strong evidence that cellular mechanisms exert a profound influence upon the organisation and orientation of matrix they deposit (Birk and Trelstad, 1984; Young et al., 2014; Koudouna et al., 2018b). However, synthesis of the initial primary stroma has been less studied and evidence for mechanisms controlling fibrillogenesis are less clear. Primary stromal fibrils are deposited close to the sub-epithelial basal lamina and early ideas that changes in fibril orientation appear to be more likely controlled by molecular factors inherent in interacting matrix components have not been challenged (Trelstad and Coulombre, 1971). Similarly, angular shifts in fibril orientation may also be less likely regulated directly through cellular mechanisms than as a result of molecular interactions.

Developments in imaging technology that permit three-dimensional observation at high resolution (Denk and Horstmann, 2004; Knott et al., 2008), can provide new insights into events during assembly of the primary stroma. Previously, we used serial block face scanning electron microscopy (SBF SEM) to examine the secondary stroma of the embryonic chick mid-way through development enabling an appreciation in three dimensions of a system of extended cell processes that aligned with emerging collagen fibril bundles (Young et al., 2014). Here, we use SBF SEM to investigate the earliest developmental stages in the sequence of matrix formation in the chick cornea, which suggests involvement of a self-directed mechanism of collagen assembly rather than a cell-directed one, that is believed to dominate later developmental stages. We also describe three-dimensional characterisation of a population of extracellular matrix cords that link the epithelial basement membrane with subjacent neural crest cells and present a model speculating how they might serve a mechanical role in the development of corneal curvature.

## 2 Materials and methods

### 2.1 Tissue acquisition

Fertilized white chicken eggs were obtained from a commercial hatchery (Henry Stewart, Louth, UK) and incubated at 37.8 °C and ~60% humidity. Developing embryos were treated in accordance with the Association for Research in Vision and Ophthalmology Statement for the Use of Animals in Ophthalmic and Vision Research and with the approval, under schedule 1, of the UK Government's Animals (Scientific Procedures) Act 1986. Table 1 summarises the numbers of embryos used, plus specimens derived and imaged by SBF SEM in respect of the different stages of embryonic development studied.

**Table 1:** Summary of chick embryo specimens used for SBF SEM.

Stage	Total Embryos	Total specimens	No. Specimens imaged	SBF SEM datasets	Total Images
E3	12	16	2	2	517/580
E4	28	48	8	10	422/919/600/431 452/398/192/532/120/1000
E5	4	8	3	5	900/701/372/630/1000
E6	19	27	5	7	782/1000/561/408/613 1000/517
E7	4	6	2	2	580/1000
E8	4	6	1	2	444/677
E10	4	8	3	4	561/1000/1000/1000
E14	13	26	2	3	455/560/1000

In some embryos, the lens was removed from the right eye at E3 as described previously (Lwigale and Bronner-Fraser, 2007) after which development was allowed to continue until E4 or E6, with the left eye serving as a control. Numbers of specimens used for this part of the study are shown in Table 2. All procedures carried out in this work comply with the ethical standards of the relevant national and institutional guides on the care and use of laboratory animals.

**Table 2:** Summary of tissue samples for Lens ablated eyes.

Total embryos ablated at E3	Stage taken	Total specimens	No. Specimens imaged	SBF SEM datasets	Total Images
6	E4 3	3 control	1	2	159/1000
		3 ablated	1	1	889
	E6 3	3 control	0	0	0
		3 ablated	2	2	530/720

### 2.2 Tissue processing

Dissected corneas, or whole eyes, from embryos at 3–14 days of development were fixed in 0.1 M sodium cacodylate-buffered 2% paraformaldehyde/2.5% glutaraldehyde at pH 7.2 for 3 h. Early stage embryos (E3-E4) were isolated using a filter paper template, as described by Chapman et al. (2001), and transferred to fixative. After rinsing and storage at 4 °C in the same buffer, specimens were processed by a modification of the method described by Deerinck et al. (2010) for enhancing tissue backscatter electron contrast for SBF SEM. Briefly, they were immersed sequentially in aqueous solutions of 1.5% potassium ferricyanide/1% osmium tetroxide, 1% thiocarbohydrazide, 1% osmium tetroxide, 1% uranyl acetate and finally lead aspartate, each for 0.5–1 h, followed by washing with distilled water. They were then dehydrated in ethanol and embedded in epoxy resin, prior to sectioning on a UC6 ultramicrotome (Leica Microsystems, Vienna, Austria). Semi-thin (2–3 μm thick) sections were collected on glass slides and stained with toluidine blue for bright field light microscopy. Ultrathin sections (70–100 nm thick) were cut for transmission electron microscopy. The polished tissue block surface was then imaged in a Sigma VP FEG scanning electron microscope (Carl Zeiss Ltd, Cambridge, UK), at 3.5 Kv, 25 Pa nitrogen pressure, 8 msec dwell time and magnification of 4.16 kx. The scan resolution was 4096 x 4096 pixels, with a resolution of 10 nm/px. Sequences of around 1000 serial images were recorded, each alternating with removal of a 100 nm surface slice by an in-chamber 3View®2 ultramicrotome (Thermo Fisher Scientific-Gatan Inc, Pleasanton, CA).

### 2.3 Proteoglycan localisation

Proteoglycans were imaged by transmission electron microscopy in additional specimens, summarised in Table 3, prepared by cationic dye contrast-enhanced fixation using the critical electrolyte concentration technique of Scott (1980). Dissected blocks of cornea were fixed for 18 h in 2.5% glutaraldehyde in 25 mM sodium acetate, at pH 5.7, containing 0.1 M magnesium chloride and 0.05% cupromeronic blue. After washing in buffer and immersion in aqueous 1% sodium tungstate, specimens were then passed through 1% sodium tungstate in 50% ethanol, before dehydration in ethanol and embedding in the same way as specimens prepared for SBF SEM.

**Table 3:** Summary of tissue samples for PG localisation with Cupromeronic blue

Stage	Total embryos	Total specimens	No. specimens imaged	TEM Images
E4	3	6	2	17
E6	3	6	2	12
E10	3	6	2	17

### 2.4 Image analysis and modelling

3-dimensional reconstructions of tissue architecture were generated using the 3D Viewer plug-in in ImageJ/Fiji, or using Amira 6.2 image analysis software. The Volume Viewer plug-in in ImageJ/Fiji was used to examine collagen fibril orientations at different depths in the primary stroma after adjusting the image plane for observations in the z direction, corresponding to epithelium towards endothelium.

## 3 Results

### 3.1 Morphogenesis of the primary stroma in three-dimensions

**Fig. 1:**
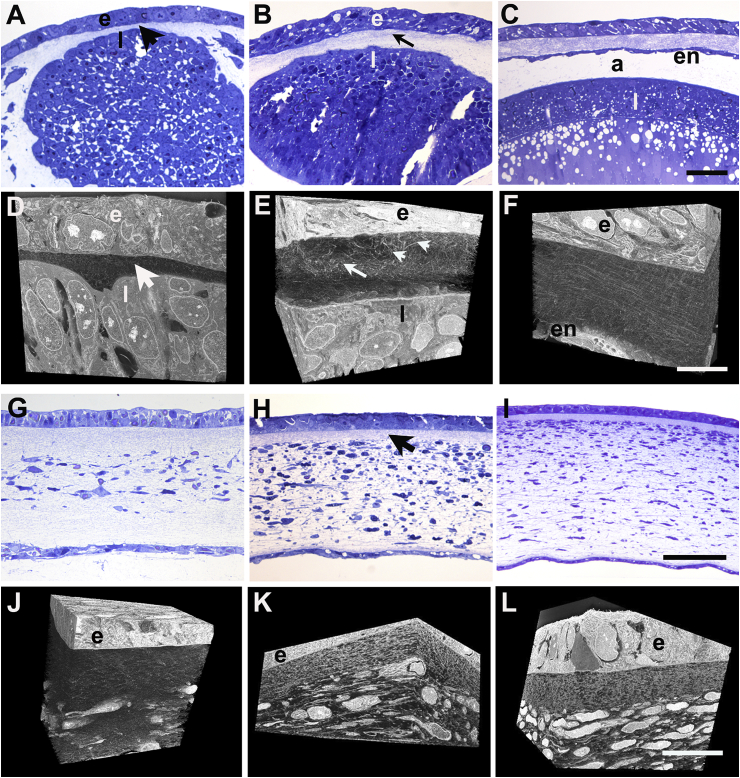
Corneal development in embryonic chick. Light microscopy of toluidine blue-stained semi-thin sections and corresponding three-dimensional reconstructions from SBF SEM at E3 (A, D), E4 (B, E), E5 (C, F), E6 (G, J), E7 (H, K) and E8 (I, L). At E3, the lens (l) is separated from ectoderm (e) by a thin layer of primary stroma (arrowhead) (A, D). At E4 a multi-layered epithelium (e) overlies a thickened primary stroma with a central undulating fibril condensation (arrow) plus fibrils at large angles to epithelial basement membrane (arrowheads) (B, E). At E5 an endothelial monolayer (en) covers the proximal face of the primary stroma, which shows more parallel arrangement of fibrils; further separation of the lens gives rise to the anterior chamber (a) (C, F). At E6 invading periocular neural crest cells populate the full width of the central region of the swollen primary stroma (G). By E7 cells occupy the whole depth of the stroma and secondary stromal biosynthesis is underway, with only a small extent of sub-epidermal acellular primary stroma remaining (arrow, H). By E8 secondary stroma synthesis increases overall thickness of the stroma and cells appear more flattened (I). In reconstructions (J–L), endothelium is not included in field of view, which shows epithelium (e), sub-epithelial primary stroma and stromal cells. At E7 keratocytes populate the stromal matrix, except for a narrow zone of residual primary stroma below the epithelium. Bar, 50 μm, A - C and G - I; 10 μm, D - F and J - L. (For interpretation of the references to colour in this figure legend, the reader is referred to the Web version of this article.)

Primary stroma was first detected at E3 as a layer, around 15 μm thick, of loosely-distributed collagen fibrils located between surface ectoderm and the forming lens (Fig. 1A and D). By E4 collagen distribution appeared inhomogeneous, with a central condensation of fibrils, which from some aspects undulated regularly with respect to the epithelial basal lamina, and which was situated between more-sparsely dispersed fibrils distally and proximally (Fig. 1B and E). Some fibrils orientated at large angles to the basal lamina were also present. Fibril organisation developed a more stratified appearance from E5 at which time the primary stroma was surfaced proximally by a complete monolayer of endothelium and was separated from the lens by the developing anterior chamber (Fig. 1C and F). Between E5 and E6, the primary stromal thickness increased to approximately 100 μm and mesenchymal cell infiltration was evident (Fig. 1G). Cells had migrated inward from the limbal region to form a continuous cellularized zone across the diameter of the mid primary stroma by E6, with un-colonised, acellular, distal and proximal layers (Fig. 1G and J). At E7, the full thickness of the developing stroma was occupied by cells, except for a narrow region immediately below the epithelial basement membrane (Fig. 1H and K), which persisted as stromal thickness increased at E8, and after (Fig. 1I and L).

### 3.2 Collagen fibril orientation in primary stroma

**Fig. 2:**
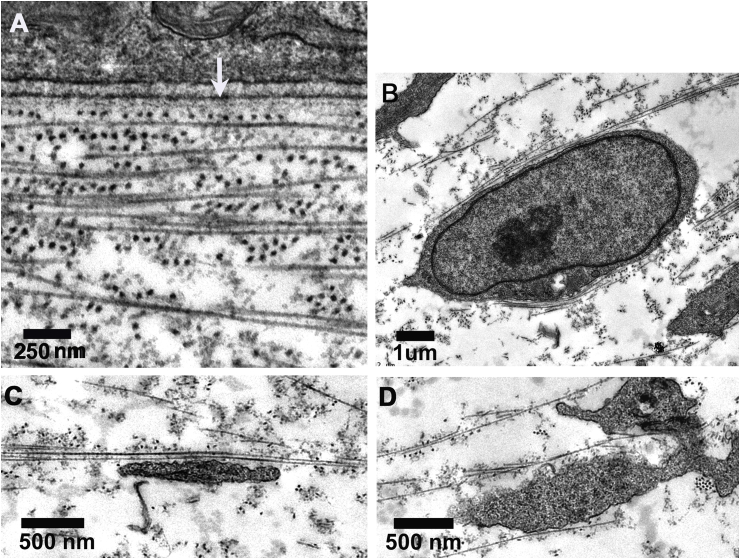
Transmission electron microscopy of the corneal primary stroma at E6. Orthogonality between adjacent micro-lamellae is evident in the immediate sub-epithelial stroma (A), with fibrils appearing as dark circles running perpendicular to the image plane and as long thin structures running within the image plane. Epithelial basal lamina is indicated by white arrow. Cells and cell processes within the primary stroma are closely apposed to collagen fibrils (B–D).

From transmission electron microscopy images, the average centre-to-centre separation between adjacent fibrils in an E6 cornea measured 69.7 ± 12 nm (SD), while the radius of the fibrils was 17.0 ± 2 nm (SD). Collagen fibrils immediately juxtaposed to the epithelial basal lamina appeared in thin section to form ‘micro-lamellae’, just 1–3 fibrils thick, with orientation alternating precisely by 90° (Fig. 2A). At early E6 when cells first invade the primary stroma, cells and processes appeared aligned and flattened with respect to these micro-lamellae (Fig. 2B–D).

**Fig. 3:**
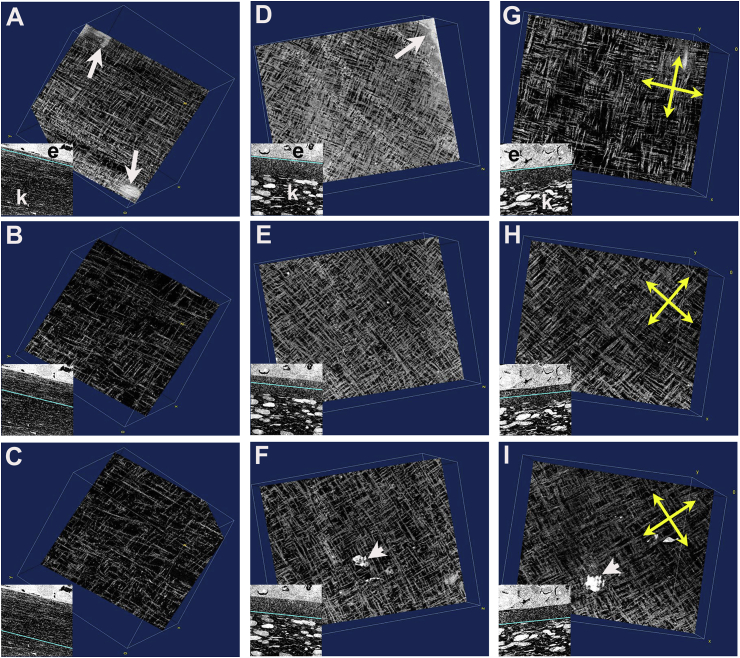
Image sequences from SBF SEM datasets show fibril orthogonality using ImageJ/Fiji, Volume Viewer plug-in. En face sequences run vertically, distal-towards-proximal views are presented at early E6 (A–C); E7 (D–F) and E8 (G–I). Collagen fibril organisation is seen at sites in sub epithelial (A, D, G), mid stromal (B, E, H) and deep proximal primary stromal matrix (C, F, I). Insets show the corresponding level in sagittal plane (line, in cyan) within the primary stroma from which the associated main image derives, showing epithelium (e) and stromal cells (k). Thus at subepithelial levels, basal epithelium is occasionally visible (arrows: A, D) and processes of stromal cells (arrowheads: F, I) in deep primary stroma. Compared to E6, at E7 and E8, the extent of acellular primary stroma is much reduced. Clockwise rotation of fibril orientation is evident in E7 and E8 sequences (indicated by yellow arrows in E8, G - I). (For interpretation of the references to colour in this figure legend, the reader is referred to the Web version of this article.)

SBF SEM datasets, viewed in ImageJ/Fiji via the Volume Viewer plug-in, permitted collagen fibril orientation and orthogonality to be observed directly in a plane parallel to the epithelial basement membrane and at manually selected depths throughout the primary stroma (Fig. 3). Datasets were manipulated such that the z-direction through the dataset corresponded with traversing the primary stroma in a distal (epithelial) to proximal (endothelial) direction. A distinctive orthogonal arrangement of fibrils was marked in the primary stroma of all stages examined from E5 through to E14. Fibril orthogonality was evident immediately below the basal lamina at each stage, except E14 at which point the typical meshwork arrangement of fibrils characteristic of Bowman's layer, the most superficial layer of the corneal stroma, was present (not shown). At E6 fibril orthogonality was preserved in mid and deep primary stroma, notwithstanding the swelling of the stroma at this stage, which was reflected in a clear reduction in fibril density (Fig. 3A and C).

Fourier transforms of z-slices of the SBF SEM reconstructions were conducted after a series of spatial rotations that brought the plane of the presumptive lamellae to coincide with the x-y plane of the reconstruction. This revealed that the Fourier transform of the parallel collagen fibrils was formed of linear lobes of intensity perpendicular to the main axis of the fibrils, passing through the centre of the transform. If the fibrils make an angle with the x-axis, α degrees, the lobe subtends an angle equating to 90+α degrees. Accordingly, collagen fibrils in real space and in the streak in the Fourier transforms make the same angle with the x-axis (minus the 90° constant). In our analysis the Fourier transform presents two orthogonal lobes of intensity passing through the centre of the transform, confirming orthogonality of collagen fibrils in adjacent stromal micro-lamellae (Supplementary Material Videos 1 and 2 for E6 and E8, respectively). Notable in our analyses is a conspicuous shift in the orthogonal array of collagen fibrils at stages E7 (Fig. 3D–F), E8 (Fig. 3G–I) and thereafter (not shown), with a progressive clockwise rotation of the main axes with increasing depth.

Supplementary video related to this article can be found at https://doi.org/10.1016/j.exer.2019.107772.

The following is/are the supplementary data related to this article:

video 3 video

video 4 video

### 3.3 Proteoglycan-collagen associations

**Fig. 4:**
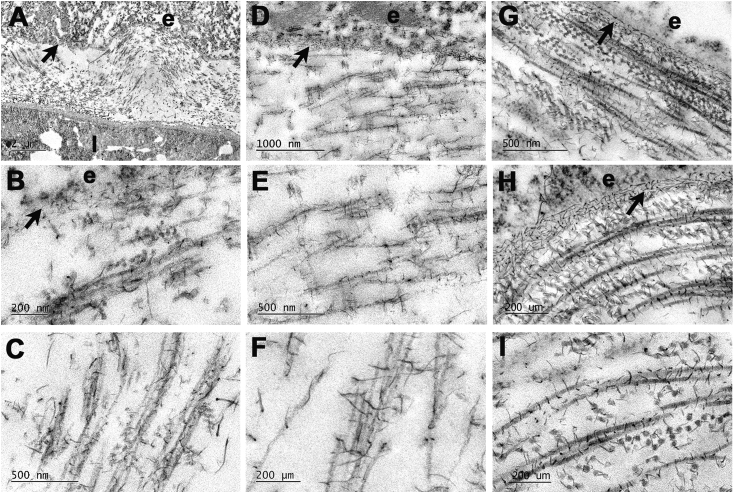
Transmission electron microscopy of primary stroma in E4 (A–C), E6 (D–F) and E10 (G–I) chick cornea after contrast enhanced fixation of proteoglycans with cupromeronic blue. Whole thickness stroma is seen in A; sub-epithelial stroma in B, D, G and H, and mid-depth stroma in C, E, F and I. Epithelium (e) and lens (l) are separated by undulating primary stromal collagen at E4 (A). Epithelial basement membrane (arrow) exhibits a complex array of proteoglycans as dense filaments at all stages. Proteoglycan filaments, regularly distributed along collagen fibrils are evident at E4 and E6, including many elongate elements traversing fibril bundles and running axially along fibrils (C, F). At E10, proteoglycan filament-collagen periodic alignment and linkages between fibrils in longitudinal and transverse section are evident (G–I).

Transmission electron microscopy of developing corneas fixed in the presence of the cationic dye, cupromeronic blue revealed proteoglycans as elongate, electron-dense filaments (Fig. 4). Complex arrays of proteoglycan filaments were associated with the epithelial basement membrane (E4: Fig. 4A and B; E6: Fig. 4D; E10: Fig. 4G and H). The majority of filaments showed regular distribution along collagen fibrils with a periodicity corresponding to that of the fibril axial banding pattern. In addition, more elongate filaments were present, traversing fibril bundles and running axially along fibrils (Fig. 4C and F). Proteoglycan filament-collagen periodic alignment and linkages between fibrils in longitudinal and transverse section were evident at E4 (Fig. 4A–C), E6 (Fig. 4D–F) and E10 (Fig. 4G–I).

### 3.4 Matrix cords

**Fig. 5:**
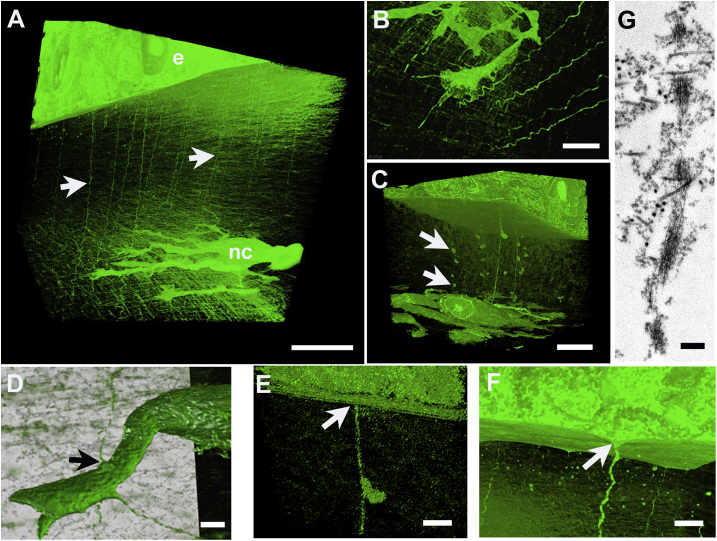
Three-dimensional reconstructions and ultrastructure of matrix cords in developing stroma of embryonic chick cornea. At late E5 (+22 h) cords (arrows) extend from epithelium (e) into vicinity of migrating neural crest cells (nc) (A, B). Globular matrix beads (arrows, C) appear attached to cords. Matrix cords are in contact with migrating cells (arrow, D), in this case a prospective keratocyte, and are continuous with epithelial basal lamina (arrow, E), which frequently exhibits conical protrusion into subjacent stroma (arrow, F). Transmission electron microscopy shows microfibrillar substructure of matrix cord (G). Bar, 10 μm: A, C; 5 μm, B; 2 μm: D - F; 250 nm, G.

Numerous sinuous, cord-like matrix structures, originating at the epithelial basal lamina, were present extending throughout the primary stroma and deep into the cellularized stroma between E4 and E10 (Fig. 5). At E4, analysis at high resolution of selected image sequences from SBF SEM datasets suggested cords were continuous between epithelial basal lamina and the lens surface (not shown). At late E5, they were present both in the vicinity of cells migrating into the primary stroma and in regions where migrating cells had not yet reached (Fig. 5A and B and Supplementary Material Video 3). Cords were found to be in contact with cytoplasmic processes of invading cells – prospective endothelium at E5 (not shown) and keratocytes at E5 - 6 (Fig. 5D). Three-dimensional reconstructions clearly showed cords to be attached to the basal lamina of the epithelial basement membrane (Fig. 5C and E). On occasion, matrix cords associated with globular structures with basal lamina-like appearance (Fig. 5C and E), and frequently appeared continuous with a conical projection of the adjacent basal cell (Fig. 5F). Transmission electron microscopy confirmed that matrix cords were not membrane-bound, but consisted of groups of parallel microfibrils, which often ran in close proximity to collagen fibrils throughout the stroma (Fig. 5G).

Supplementary video related to this article can be found at https://doi.org/10.1016/j.exer.2019.107772.

The following is/are the supplementary data related to this article:

video 5 video

**Fig. 6:**
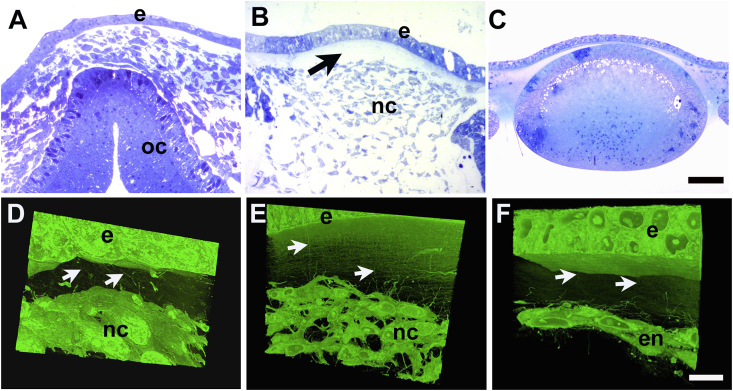
Corneal morphogenesis after ablation of the lens at E3. Light microscopy of toluidine blue-stained sections, top, and three-dimensional models generated in Amira software, below. Ablated eye at E4 showing corneal epithelium (e) and neural crest cell mass overlying closed optic cup (oc) (A, D). In an ablated eye at E6, primary stroma (arrow, B) is present below epithelium (e), but neural crest cells have failed to form endothelial monolayer, instead a central mass of disorganised cells (B, nc). A non-ablated control eye at E4 exhibits normal development of lens and early corneal primary stroma (C). In ablated eye at E4 (D) and E6 (E) matrix cords (arrows) are evident extending from epithelium (e) into disorganised neural crest cell mass (nc) proximally. In an unablated eye, at E5 neural crest cells have formed almost complete endothelial cell (en) monolayer on distal face of primary stroma, where matrix cords are evident (arrows, F). Bar, 50 μm: A - C; 10 μm: D - F. (For interpretation of the references to colour in this figure legend, the reader is referred to the Web version of this article.)

To determine whether matrix cords were dependent on the presence of the lens and/or cellular organization of the corneal endothelium and keratocytes, we performed lens ablation prior to the invasion of periocular neural crest cells at E3. In eyes subjected to lens ablation, a disorganised accumulation of mesenchymal cells was present at E4 and E6 (Fig. 6A, B, 6D and 6E and Supplementary Material Video 4), instead of the organised migration of formative endothelium (Fig. 6F) and keratocyte populations in un-operated eyes. However, a rudimentary primary stroma was synthesised in which matrix cords were evident (Fig. 6D and E), suggesting that the lens is not required for their presence, and that they are not sufficient to support the formation of a corneal endothelium from the first wave of neural crest invasion.

Supplementary video related to this article can be found at https://doi.org/10.1016/j.exer.2019.107772.

The following is/are the supplementary data related to this article:

video 6 video

## 4 Discussion

Major questions still exist concerning the nature of mechanisms governing the assembly of the ordered collagen matrix of the corneal stroma. Our previous investigations into the synthesis of the secondary chick stroma, which is fully populated by keratocytes from E12 onwards, allowed us to characterise cell-cell and cell-matrix interactions in three-dimensions (Bushby et al., 2011) and provided evidence for *cell-directed* matrix deposition, because the long axes of the extensive network of keratocyte cell processes corresponded with the alignment of the emerging fibril bundles (Young et al., 2014). Earlier in development, however, the striking orthogonality of fibrils deposited in micro-lamellae below the epithelial basement membrane, in a region of the tissue devoid of presumptive keratocytes, suggests that molecular interactions may play a part in determining fibril spacing and orientation. Our observations from SBF SEM at high resolution and in three dimensions imply that angular displacement of fibril orientation with depth is present even in early stages of stromal development. In the absence of stromal cells, it appears that this process may also be influenced by molecular interactions. Thus, whilst the predominant mechanism for the formation of the secondary cornea in the developing chick seems to rely upon alignment of cells, observations here, albeit from limited numbers of specimens, encourage us to consider that collagen alignment in early development may be *self-directed.*

The diameter of fibrils in the primary stroma is remarkably uniform, a characteristic shared with fibrils in mature corneal stroma. A diverse range of macromolecules has been implicated in the control and regulation of collagen fibril dimensions and order in connective tissues, including small leucine-rich proteoglycans such as decorin and biglycan (Zhang et al., 2009; Robinson et al., 2017), lumican, keratocan and fibromodulin (Chakravarti et al., 2000; Quantock et al., 2001; Liu et al., 2003; Meek et al., 2003; Chen et al., 2010), as well as minor collagens, including type V, type XII and XIV (Young et al., 2002; Sun et al., 2011; Chen et al., 2015). However, molecular heterogeneity is thought to be far less diverse in the earliest stages of primary stroma synthesis than at later stages, when the stroma is invaded by mesenchymal cells (Linsenmayer et al., 1998), and the nature of molecular interactions might therefore be expected to be less complex. Unsulphated and sulphated chondroitin sulphate/dermatan sulphate (CS/DS) proteoglycans predominate at E5 with keratan sulphate proteoglycans increasing thereafter and thought to be synthesised by cells in the stroma (Nakazawa et al., 1995; Liles et al., 2010). Cupromeronic blue-positive material visible here by electron microscopy in E5 and E6 corneas is likely to represent largely collagen fibril-associated CS/DS proteoglycans as well as CS chains that are known to be present on type IX collagen (Bruckner et al., 1985; Irwin and Mayne, 1986).

Although fibrils in primary and mature stroma have a different chemical composition, there is an important characteristic that they have in common; both sets of fibrils are heterotypic in nature. This was hypothesised to be an important factor in maintaining fibril diameter uniformity in mature corneas, since small mismatches between the collagen molecules would affect the forces responsible for holding the molecules together and would limit the maximum number of molecules able to form a fibril (Birk et al., 1990). The same mechanism may pertain for the primary stroma, where type II collagen associates with type IX to form heterotypic fibrils. In addition, type IX collagen molecules present interrupted collagenous domains and non-collagenous domains along with a chondroitin sulphate side chain that are likely to limit the assembly of large numbers of collagen molecules together.

**Fig. 7:**
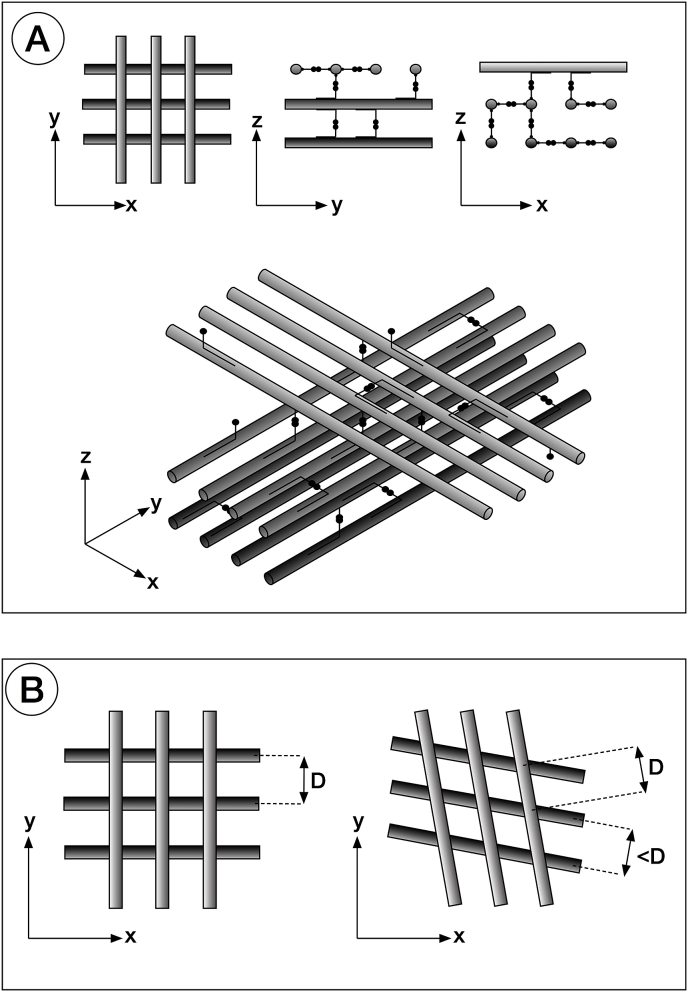
Schematic of cell-independent orthogonality of collagen fibril arrangement. Collagen fibrils, shown in grey, and type IX collagen molecules (with associated CS/DS chains), in black, are all 65 nm apart giving rise to a ‘square lattice’ appearance. Only some of the collagen IX molecules are shown. A reduction in interfibrillar spacing, along with the fact that the position of the collagen IX molecules along the collagen fibrils does not change, brings about a rotation of adjacent collagen layers with respect to each other (B).

In the primary stroma, the distance between fibrils is very similar to the axial periodicity of the fibrils themselves. We postulate that the interfibrillar distance and the fibril periodicity are in fact the same and that the discrepancy between the two measurements can be attributable to minor volume changes in processing for electron microscopy (Fig. 7A). This arrangement can be explained by the interaction of type IX collagen molecules projecting from adjacent collagen fibrils (Cai et al., 1994). Symmetry considerations suggest that collagen type II - type IX interactions within fibrils of the primary stoma must result in a periodic decoration of the surface of the collagen fibrils, with the periodicity being the same as that of the heterotypic fibrils. Because the amino-terminal domains of type IX collagen molecules project outwards with their CS chain exposed, they are free to bind to terminal domains of other collagen IX molecules. This bond may be identical to those postulated for the proteoglycans of the secondary stroma (Lewis et al., 2010), where antiparallel associations between glycosaminoglycan chains are mediated by ions present in the inter-fibrillar spaces.

Transmission electron microscopy of the sub-epithelial primary stroma at E6 indicates that uniformly thin collagen fibrils form micro-lamellae, a few fibrils thick, in which the fibril axes in adjacent lamellae are at right-angles in the plane of the cornea (Fig. 7A). The absence of any directionality in the corneal epithelial cells that synthesise these collagen fibrils leads us to reconsider the view, previously expressed (Trelstad and Coulombre, 1971), that the formation of an orthogonal array is self-directed and intrinsic to some property of the collagen fibrils. Collagen fibrils tending to assemble with random orientations in the sub-epithelial space would be compelled to re-orient themselves in such a way that all their axes become parallel and close to each other. This would be to maximise the number of bonds between collagen IX molecules on adjacent fibrils. As more fibrils are synthesised, there is a choice of possible orientation. One is to form a fibril layer with the same orientation as the existing ones; the other is to assume an orientation perpendicular to the existing fibrils. The number of interactions of collagen IX molecules on adjacent perpendicular fibrils would still be the same, provided that the distance between adjacent parallel fibrils is the same as their periodicity, and that the fibrils are arranged in layers (Fig. 7A). Orientations that are neither parallel nor perpendicular would not maximise the number of bonds between collagen IX molecules and therefore would not be energetically favourable. In this model, the role of hyaluronan and other proteoglycans in the stroma would be that of attracting water molecules and maintaining the correct level of hydrostatic pressure between fibrils to help maintain the correct interfibrillar distance.

Between E5 and E6, the primary stroma swells, potentially because of enzymatic removal of type IX collagen (Cai et al., 1994), or a switch in biosynthesis from the ‘long’ isoform of type IX with the highly interactive NH_2_-terminal domain, to the short form lacking it (Fitch et al., 1995). Mesenchymal cell invasion of the primary stroma then begins. A likely outcome would be a change in the chemical environment experienced by the fibrils as has already been shown to occur in later stages of corneal morphogenesis (Koudouna et al., 2012). The distance between the collagen fibrils could thus be established by a balance between attractive forces arising from collagen IX molecules binding together, and repulsive hydrostatic forces from charged groups on hyaluronan and other proteoglycans, ultimately attracting additional ions and water molecules via the Donnan effect. A change in chemical environment conceivably involving a change in forces experienced by the fibrils, could lead to a new equilibrium distance (D) between the fibrils (Fig. 7B). A small decrease in D, along with the fact that the crossover points (where opposing collagen IX molecules interact with each other) cannot change, since they depend on the collagen fibril periodicity, would be accommodated by a relative rotation between collagen fibrils belonging to adjacent layers (Fig. 7B). This rotation is very small. Our calculations show that at E8 there is a change in fibril orientation of about 70° for a 15 μm change in depth. If one considers a theoretical situation where fibril orientation alternates depth-wise between adjacent fibrils in this region, there are nominally about 15,000/67 ≃ 220 layers, the angle between adjacent layers is only 70/220 ≃ 0.3°. This corresponds to an almost imperceptible decrease in spacing between adjacent fibrils. The fact that fibril rotation always occurs in one particular direction may be explained by the interactions made by collagen IX molecules and their chondroitin sulphate chains. These molecules are likely to oppose more resistance if twisted along their axis in one direction, making them more likely to all twist in the opposite way. They would dictate the angle (positive or negative) between adjacent layers, thus determining the handedness of rotation throughout the entire primary stroma.

Angular displacement of fibrils, determined by molecular interactions in the primary stroma may thus, by providing a scaffold to guide orientation of migrating mesenchymal cells, influence the rotation of fibrils subsequently synthesised as the secondary stroma. Cell orientation has been shown to affect the direction of collagen fibres synthesised by cells in culture (Wang et al., 2003). A detailed recent analysis of fibril angular displacement by second harmonic generation imaging (SHG) through full stromal thickness from E8 through E18 showed a clear rotational shift from E10 onwards (Koudouna et al., 2018b). No displacement was identified at E8 or E9, we assume, because at these stages the combined diffuse nature of the collagen matrix and small diameter, around 30 nm, of the fibrils in the primary stroma is insufficient to provide a detectable SHG signal. Spatial resolution in SHG is linked to the diffraction limit associated with the wavelength of incident light which, at several hundred nanometres cannot equal that achievable by electron microscopy (Mostaço-Guidolin et al., 2017). High resolution SHG imaging of rat tail tendon generated an intensity profile from which calculations indicated a hollow tube-like signal origin with diameter of 0.2–0.3 μm, corresponding to the comparatively large size of individual tendon collagen fibrils (Williams et al., 2005). In contrast, at E9, keratocytes in the anterior 45 μm of the stroma exhibited a detectable clockwise rotation by confocal microscopy which increased significantly from then on. Thus, it seems plausible that biomechanical forces generated by the wave of mesenchymal cells migrating into the mid primary stroma may also collectively impact upon collagen fibril orientation distally. SBF SEM observations confirm that rotational displacement of collagen fibrils in the distal acellular primary stroma occurs after cells invade proximal regions of this matrix. Cells cultured on a substrate constantly probe, push and pull on the substrate via traction forces at the cell-substrate interface (Harris et al., 1980), with traction forces induced by contractility of the actino-myosin cytoskeleton. Cell shape and behaviour may too be modified by feedback via biomechanical signals from the matrix, including stiffness and structure (Petroll and Miron-Mendoza, 2015). Extension of processes from cells migrating centrally along fibril bundles may thus perturb the orthogonal arrangement between collagen fibrils in superficial layers.

The structures we refer to herein as matrix cords were identified in early studies of embryonic chick cornea, and termed ‘strands’ or ‘strings’ (Hay and Revel, 1969; Bee et al., 1988; Fitch et al., 1991; Linsenmayer et al., 1998). Considered to be phylogenetically related to sutural fibres present in the corneas of cartilaginous fish and reptiles, their function remains unknown, although roles in the acquisition of stromal transparency, in prevention of stromal swelling, or in epithelial-mesenchymal mechano-transduction have all been postulated. Matrix cords are conspicuous components of primary stroma, persisting through secondary stromal deposition in developing chick, but have not been described in embryonic cornea of mouse or human, where no primary stroma is formed. Our observations indicate that cords are present from E4, therefore preceding cell colonisation of the primary stroma and so appear not to be a product of the presumptive keratocytes. Analysis in three dimensions of sites at E4 where cords are seen continuous with protrusions of the adjacent epithelial cell suggest they are also in contact with the distal surface of the developing lens, which at this stage has not long detached from the outer ectoderm (i.e. the formative corneal epithelium). Matrix cords may therefore represent extensions of basal lamina with common origins shared by lens and epithelium, created when the lens vesicle separated from ectoderm. Although only small numbers of eyes were examined from which the lens was removed at E3, cords were present in the primary stroma at E4 and E6 and therefore do not seem to depend on an influence from the lens for their synthesis or induction. Further work is necessary to confirm this observation.

In unpublished studies by Koudouna and Ralphs matrix cords have been found to persist in abundance into E18 and therefore seem unlikely to be involved specifically in transparency development. Our SBF SEM data indicate that matrix cords attach to the corneal epithelial basement membrane and, based on focal deformations (Fig. 5F) are likely able to exert a tensioning effect directed towards the interior of the developing eye. Up to E8 the profile of the chick cornea matches that of the rest of the eye, which is growing and expanding as a spherical object and experiencing an outward pressure from within. After E8 the cornea starts to become more curved than the rest of the eye's outer tunic, causing the corneal surface to bulge. Three-dimensional reconstructions from our SBF SEM datasets indicate that matrix cords appear to link the epithelial basement membrane to keratocytes in the subjacent stroma perhaps forming tethers, consistent with a role in the emergence of the corneal bulge.

**Fig. 8:**
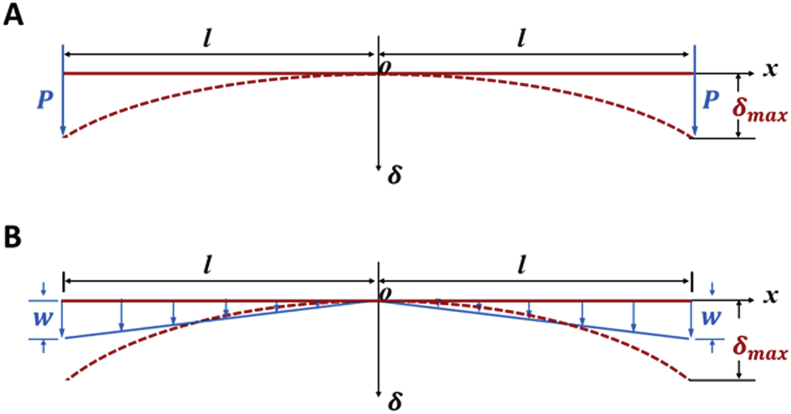
A theoretical consideration of the induction of curvature in a structure. Curvature may arise as a result of tension or load located, either: A. at the edge of the structure, or B. across its whole extent. The red dashed line indicates the deformed state caused by tension(s), which are represented by blue arrows. The solid red line indicates the non-deformed state. ***O*** represents the centre of the system, ***P*** is concentrated load, ***w*** variable load, δ the deflection at a specific point (***x***), and ***l*** the half length of the structure. (For interpretation of the references to colour in this figure legend, the reader is referred to the Web version of this article.)

As the developing chick eye grows between E8 - E14, the corneal radius of curvature is increasing. During this timeframe the *relative* radius of curvature of the cornea compared to the rest of the eye is decreasing, the cornea is becoming progressively more curved. Engineering considerations offer an explanation as to how this might happen. In a simple model of the developing cornea in two dimensions, the corneal epithelial basement membrane can be equated to a linear structure (a beam), experiencing forces from below (Fig. 8). Notwithstanding the other changes that occur in the growing eye, a tensioning or tethering force, elicited by the matrix cords on the corneal epithelial basement membrane, can be viewed as a *resultant net force*. When a beam is subjected to a *concentrated load*, *P*, at its free ends the deflection, δ, at specific point (*x*) can be described as

$$\delta =\frac{Px^{2}}{6EI}\left(3l-|x|\right)$$

where *l* is the half length of the beam (or the corneal epithelium basement membrane, in this case), *E* is the Young's modulus of the beam and *I* is the second moment of area, which is related to the dimensions of the cross section. Alternatively, the deflection, δ at specific point, *x* on the beam, when subjected to *varying load* with maximum intensity, *w* can be specified as

$$\delta =\frac{w}{120EIl}\left(20l^{3}x^{2}-10l^{2}|x{|}^{3}+|x{|}^{5}\right)$$

In both of these qualitative analyses there is a nonlinear relationship between deflection, δ and distance, *x*, so that the concept of matrix cords exerting a relative net tensioning or tethered load on the corneal surface of the developing chick eye, to induce a change in curvature, appears to be a valid one. Qualitative observations suggest that more matrix cords exist towards the periphery of the cornea, which perhaps favours the model of a concentrated peripheral load but, given that cords are seen across the whole cornea, a mixed model is perhaps more relevant.

The precise role of the primary stroma during chick corneal morphogenesis remains uncertain. It continues to be synthesised by ectodermal cells after lens ablation at E3, but no organised migration of periocular neural crest cells and subsequent formation of the endothelium, or keratocyte populations follows. This suggests that the lens may exert a more profound influence upon these developmental events than this rudimentary matrix. Its orthogonal, micro-lamellar, fibril arrangement seems to arise from cell-independent molecular interactions. Previous studies on collagen solutions *in vitro* proposed mechanisms whereby collagen fibrils might nucleate in cell-free systems. Collagen molecules can form liquid crystal assemblies (Giraud-Guille MM, 1989; Mosser et al., 2006), although little conclusive evidence has been presented for this process *in vivo*. Also, localised high concentrations of secreted collagen may be constrained by physical barriers, such as the basal lamina, and other macromolecules, as proposed by Saedi et.al., (2012), leading to molecular crowding, with the potential to promote fibril formation with orientations determined by interactions with molecules, such as type IX collagen. The primary stroma in the avian embryonic cornea will likely continue to represent an important model for studies of cell and molecular interactions in tissue morphogenesis.

## 5 Conclusions

The characteristic orthogonal arrangement of uniformly thin collagen fibrils deposited by the corneal epithelium to form the primary cornea of the embryonic chick may arise via self-directed, rather than cell-directed, mechanisms. Charge distribution along the fibril axis imparted by associated matrix components such as glycosaminoglycans, including CS/DS chains linked to type IX collagen, is one factor which may be instrumental in orchestrating the assembly process. Our three-dimensional microscopical study of non-collagenous extracellular matrix cords, which run proximally from the corneal epithelial basement membrane into the developing stroma, has demonstrated their extensive nature and widespread distribution. They are present before the second wave of neural crest cell invasion, and thus are not a product of the presumptive keratocytes. Potentially, they could serve a mechanical role in the induction of corneal curvature.

## Declaration of interest

None.
