# Supplementary material for: Cell-independent matrix configuration in early corneal development
Source: Exp Eye Res. 2019 Oct;187:107772. doi: 10.1016/j.exer.2019.107772 (PMC6892249; doi:10.1016/j.exer.2019.107772)
Supplement: Multimedia component 1 [file mmc1.docx]

**Video 1**

Fly-through of an SBF SEM image sequence from E6 cornea, along with the Fourier transform of each slice. The 326 image sequence represents a volume through the primary stroma of approximately 78 x 66 x 33 µm, where each image corresponds to a z-slice through the volume and darker shades of grey represent stained material.


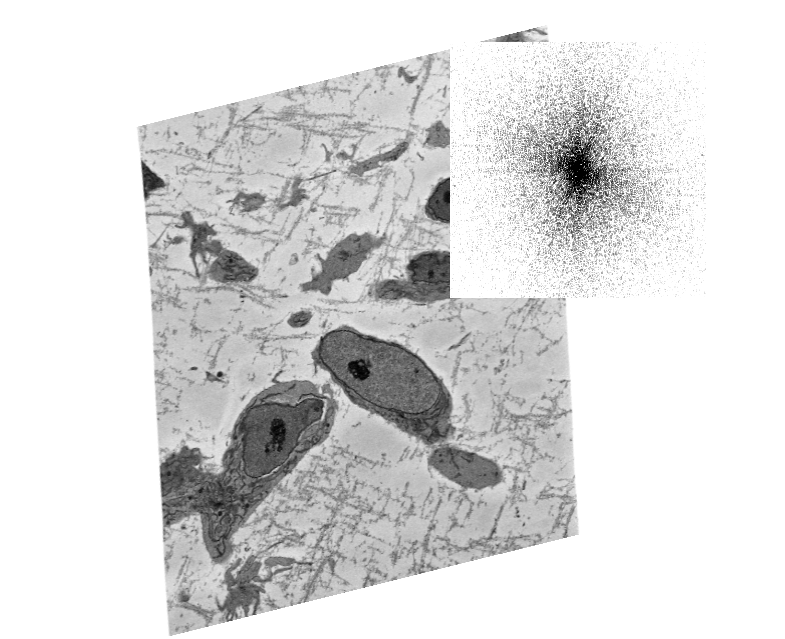


**Video 2**:

Fly-through of an SBF SEM image sequence from E8 cornea, along with the Fourier transform of each slice. The 246 image sequence represents a volume through the primary stroma of approximately 69 x 55 x 24 µm, where each image corresponds to a z-slice through the volume and darker shades of grey represent stained material.


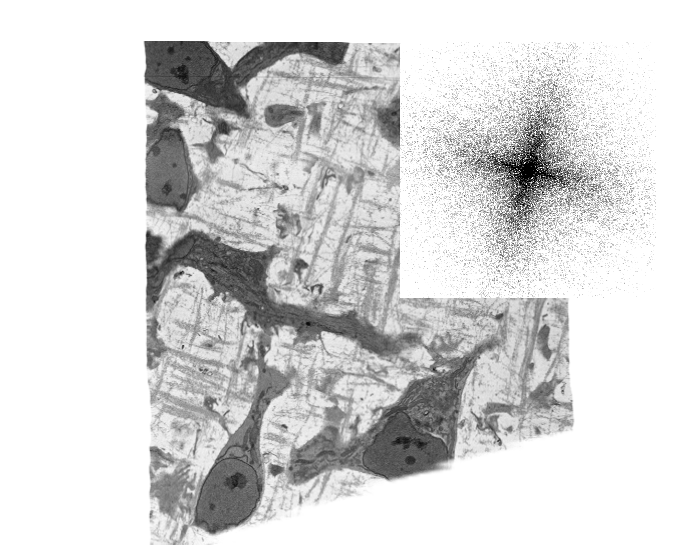


**Video 3**

3D reconstruction using Amira 6.2 software from SBF SEM images of chick cornea at E5 (+22 hr). Matrix cords extend from epithelium into vicinity of migrating neural crest cells.


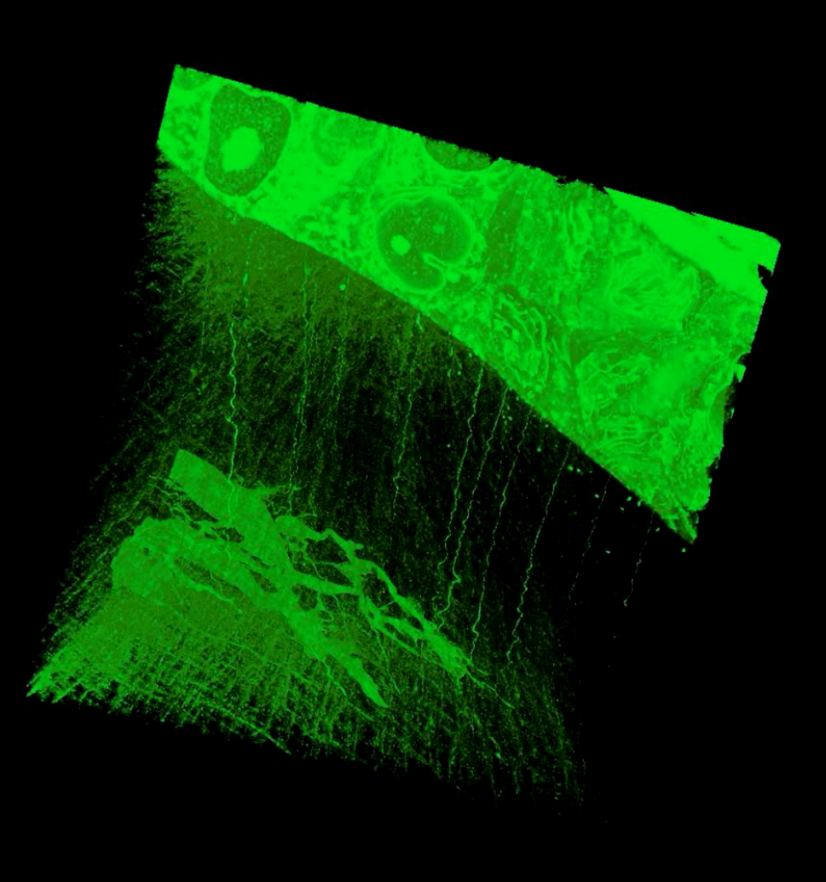


**Video 4**

Fly-through of 300 SBF SEM images from E6 embryonic chick cornea after lens ablation at E3. A disorganised accumulation of mesenchymal cells is present, instead of endothelial monolayer and keratocyte population, on the posterior face of the primary stroma, within which matrix cords can be seen.
